# Comparison of SDSG and CARDS classifications for L5/S1 lumbar degenerative spondylolisthesis: an independent inter- and intra-observer agreement study

**DOI:** 10.1186/s13018-021-02539-7

**Published:** 2021-06-15

**Authors:** Zhengwang Sun, Chongqing Xu, Mengchen Yin, Wangjun Yan

**Affiliations:** 1 Department of Musculoskeletal Oncology, Fudan University Shanghai Cancer Center, Shanghai, China; 2 Department of Orthopedics, LongHua Hospital, Shanghai University of Traditional Chinese Medicine, Shanghai, China

**Keywords:** Lumbar degenerative spondylolisthesis, SDSG classification, CARDS classification, Reliability, Agreement study

## Abstract

**Background:**

Lumbar degenerative spondylolisthesis (DS) has been a common disease that makes increasing patients to suffer from different degrees of low back pain and radicular symptoms. The Spinal Deformity Study Group (SDSG) and the Clinical and Radiographic Degenerative Spondylolisthesis (CARDS) systems are commonly used to classify the disease, and help to make a more detailed treatment plan. The objective of this study is to compare the reliability and reproducibility of SDSG and CARDS classifications, and to explore their clinical application value.

**Methods/design:**

All 117 patients with L5/S1 lumbar DS were enrolled. Five experienced spine surgeons were selected to assess DS with SDSG and CARDS systems. Kappa (K) value was used to check the coefficient consistency for multi-factor and assess the inter- and intra-observer agreement. After 12 weeks, the analysis was repeated.

**Results:**

The inter-observer reliability and intra-observer reproducibility of SDSG system were substantial with K values of 0.704 and 0.861, while those of CARDS system were substantial with values of 0.620 and 0.878.

**Conclusion:**

SDSG system had better inter-observer reliability in comparison with CARDS system, and though CARDS system is more intuitive and simpler, it is more likely to produce deviations when using it. Both SDSG and CARDS systems show substantial agreement and have great significance in surgical strategy of L5/S1 lumbar DS, they can be widely used in clinical practice.

## Background

Lumbar degenerative spondylolisthesis (DS) is defined as anterior displacement of one vertebra over the subjacent vertebra caused by degenerative changes, without an associated disruption of defect in the vertebral ring, which is mainly manifested as lumbosacral pain, sciatic nerve involvement, and intermittent claudication. It is a common disease that mostly occurs in L4/5 and L5/S1 segments. Increasing patients are suffering from different degrees of low back pain and radicular symptoms [1]. However, the pathogenesis, symptoms, and imaging manifestations often differentiate in individuals, so treatment strategies remain controversial [2, 3]. In the past decades, relevant classifications of the disease have emerged. Previously, lumbar DS was classified according to etiology and slip grade, which provided limited clinical value in guiding surgical treatment since the degree of slip rarely exceeds 30% [4, 5]. In addition, classification such as Meyerding system [6] did not consider morphological parameters related to clinical outcomes, for example, disk height or spinopelvic balance. Thus, an appropriate classification of lumbar DS is essential. Use of an appropriate classification is crucial to guide the surgical decision.

With the further study of spine biomechanics and sagittal balance, the understanding of lumbar DS pathogenic factors and natural history has been increasingly comprehensive. Nowadays, the correlation between pelvic incidence (PI) and morbidity of lumbar DS, as well as between spine sagittal balance and progression of lumbar DS has been clarified [7].

**Fig. 1:**
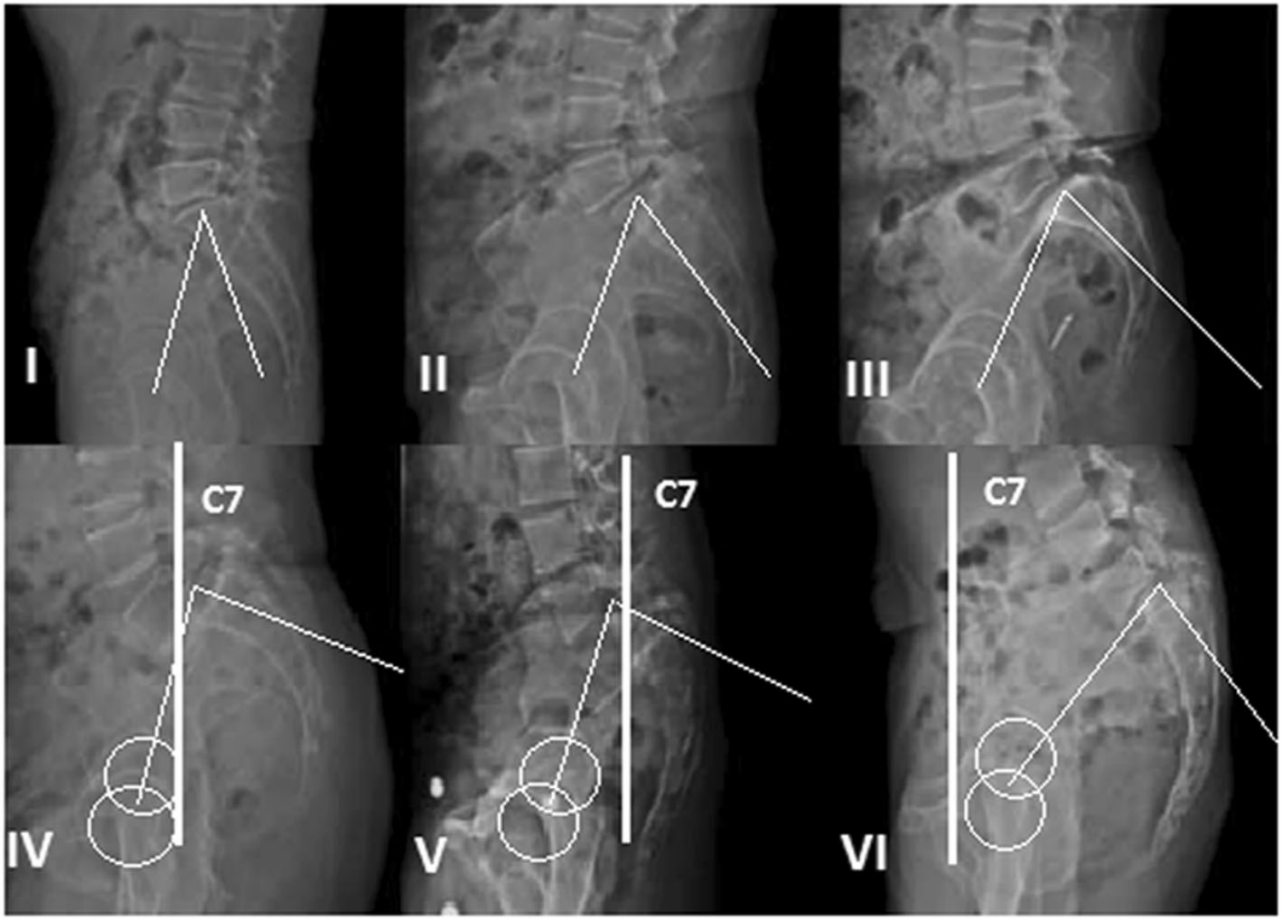
Spinal Deformity Study Group (SDSG) classification of lumbosacral spondylolisthesis. Low-grade spondylolisthesis: type 1 (low PI), type 2 (normal PI), and type 3 (high PI). High-grade spondylolisthesis: type 4 (balanced sacro-pelvis), type 5 (retroverted sacro-pelvis with balanced spine), and type 6 (retroverted sacro-pelvis with unbalanced spine)

Based on radiographic measurement of slip grade, PI, sacral slope (SS), pelvic tilt (PT), and spinopelvic balance, the Spinal Deformity Study Group (SDSG) developed a classification in 2011. Three types of low-grade spondylolisthesis are described: low PI (type 1), normal PI (type 2), and high PI (type 3). High-grade spondylolisthesis are defined as type 4 (balanced sacro-pelvis), type 5 (retroverted sacro-pelvis with balanced spine), and type 6 (retroverted sacro-pelvis with unbalanced spine) [8]. They suggested that for patients with balanced pelvis and spine, fusion can be performed either in situ or in reduction and fixation, while for those with unbalanced pelvis or spine, reduction should be emphasized in order to restore sagittal balance and provide a better biomechanical environment for fusion (Fig. 1).

**Fig. 2:**
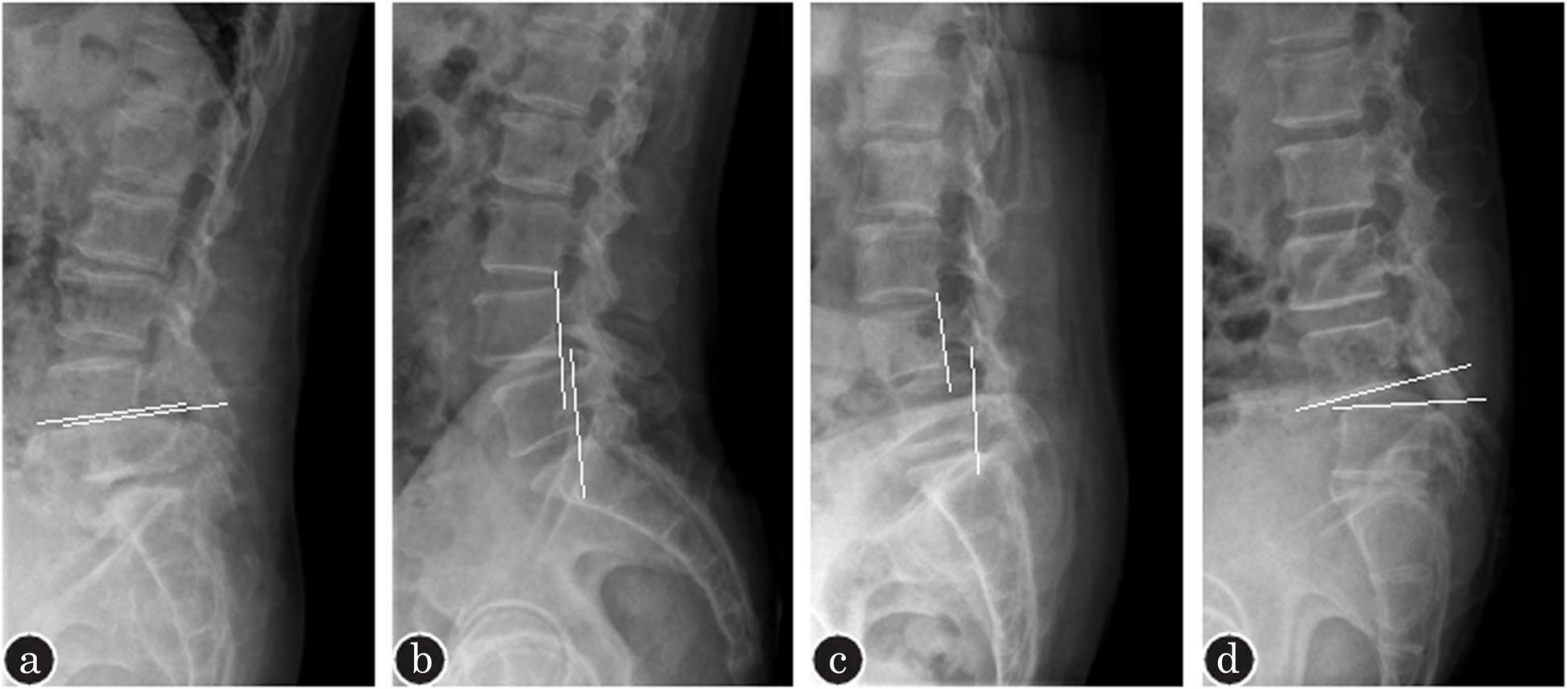
Diagram of CARDS classifications. **a** Type A, advanced collapse of L4/5 disk space and contact of adjacent endplates. **b** Type B, disk space partially preserved wish translation < 5 mm. **c** Type C, vertebral translation > 5 mm. **d** Type D, kyphotic alignment of L4/5

In 2014, Kepler et al. [9] proposed the Clinical and Radiographic Degenerative Spondylolisthesis classification (CARDS) on the basis of disk space height, sagittal vertebral translation, and kyphotic alignment. It included 4 morphologic types (A, B, C, and D) and 3 leg pain modifiers (0, 1, and 2), resulting in 12 subgroups: Types A0, A1, A2, B0, B1, B2, C0, C1, C2, D0, D1, and D2. This classification takes both radiographic parameters and clinical manifestations into consideration, so as to provide a more comprehensive evaluation for surgical treatment (Fig. 2).

However, any classification being widely used in clinical evaluation and treatment strategy decision-making should allow communication and easier consultation among specialists and needs multiple validations. The purpose of this study is to compare the inter- and intra-observer agreement of the SDSG and CARDS systems for classifying L5-S1 lumbar DS, and to compare their clinical application value.

## Materials and methods

### Patient case selection and evaluation

The study was conducted in accordance with the principles of the Declaration of Helsinki, and obtained institutional review board approval from our ethics committee. Database records of patients with L5/S1 lumbar DS treated in our hospital were retrospectively collected and analyzed between January 1, 2017, and December 31, 2019. Patients included in the study should have performed posteroanterior and lateral standing radiographs of the entire spine and pelvis showing both femoral heads (including flexion and extension lumbar position). Exclusion criteria were patients with a history or clinical signs of hip, pelvic, or lower limb disorders, and incomplete clinical data or imaging studies. All subjects were required to have complete and available clinical data including demographic characteristics, chief complaint, neurological function, complications, and treatment history.

Two physicians who collected the cases and treated these patients did not participate in the later statistics and analysis. Another 5 spine surgeons volunteered to be the observers as they were unaware of the patients’ identification, treatment history, and original classification used in clinical care. Each evaluator was provided with essential original literature and pertinent information of cases for assessment [8, 9]. Face-to-face meetings and evaluation sessions were performed before the agreement study and through which any controversies about the two classifications were discussed until all the observers came to a consensus. Standard imaging reports were available to observers as reference. Each observer used IDC Cygnus Version 1.2 (DICOM image viewing software) for calculating parameters which were needed to classify spondylolisthesis. According to the mentioned classifications, observers respectively assigned each case with a SDSG type and a CARDS type (e.g., SDSG: Type 1; CARDS: A).

Inter-observer reliability was assessed by comparing the initial responses of the 5 observers. The intra-observer reproducibility was determined through a comparison between the two responses which were separated by a 12-week interval, and all cases in the first and second acquisition sessions were displayed randomly so as to minimize the recall bias.

### Statistical analysis

All data analyses were performed using Stata Version 16.0 (software for statistics and data science). Considering that the classifications of SDSG and CARDS systems belonged to ordinal data, we used Fleiss’s kappa (K) coefficient as well as percentage of agreement to assess inter-observer reliability, while intra-observer reproducibility was evaluated based on the first and second acquisition session for each observer by Cohen’s K coefficient and percentage of agreement [10, 11]. The K values were expressed with a 95% confidence interval (CI), and the range of the coefficient is between −1 and 1. Higher values signified better agreement. According to the study by Landis and Koch [12], levels of agreement for K were divided into five grades, with κ values 0.00 to 0.20 considered as slight; 0.21 to 0.40, fair agreement; 0.41 to 0.60, moderate agreement; 0.61 to 0.80, substantial agreement; and 0.81 to 1.00, near perfect agreement. Meanwhile, *p* values of < 0.05 were considered statistically significant for all the above.

## Result

This study totally involved 117 consecutive patients, including 45 males and 72 females, with an average age of 66.3 years (range from 52 to 84 years). All 6 types of SDSG system and 12 types of CARDS system were obtained within those individuals. There were 1170 evaluations made by 5 observers using SDSG classification in 2 assessments (117*5*2), including type 1 (17.1%), type 2 (30.8%), type 3 (22.2%), type 4 (12.8%), type 5 (10.3%), and type 6 (6.8%). Similarly, 1170 evaluations of CARDS classification were assigned to 15.4% type A (A0, 5; A1, 121; A2, 54), 42.7% type B (B0, 0; B1, 317; B2, 183), 36.8% type C (C0, 0; C1, 294; C2, 136), 5.1% type D (D0, 0; D1, 23; B2, 37).

### Inter-observer reliability

The overall inter-observer agreement of SDSG classification was substantial at 79.4% (74.4-85.5%) with an associated K value of 0.704 (0.655-0.769) (Table 1), of which 58 (49.6%) cases showed perfect agreement among all 5 observers in the first assessment, while 61 (52.1%) cases in the second assessment. In addition, at least 3 observers showed agreement on 101 (86.3%) cases in the first assessment and 103 (88.0%) cases in the second assessment (Table 2). Besides, the inter-observer agreement of slip grade (low-grade vs. high-grade slips) was near perfect at 89.2% (85.1-91.7%), with a k value of 0.813 (Table 3).

**Table 1:** Inter-observer reliability of SDSG classification

Observers^a^	Cases in agreement between observers							Agreement (%)	K
	Type 1	Type 2	Type 3	Type 4	Type 5	Type 6	Total		
1-2	15	27	19	11	9	6	87	74.4	0.655
1-3	16	30	19	12	12	6	95	81.2	0.728
1-4	15	28	21	10	11	5	90	76.9	0.671
1-5	17	30	18	12	11	6	94	80.3	0.713
2-3	14	29	21	13	12	6	95	81.2	0.727
2-4	18	32	20	11	13	6	100	85.5	0.769
2-5	15	29	20	11	11	6	92	78.6	0.694
3-4	15	28	19	12	12	6	92	78.6	0.693
3-5	16	28	20	11	12	6	93	79.5	0.707
4-5	14	29	19	12	11	6	91	77.8	0.682
Overall	-	-	-	-	-	-	-	79.4	0.704

^a^1, 2, 3, 4, 5 represent the 5 observers who participated in the study

**Table 2:** Agreement of SDSG classification among five observers

	The first assessment	The second assessment
**Agreement among all 5 observers**	58 (49.6%)	61 (52.1%)
**Agreement among at least 3 observers**	101 (86.3%)	103 (88.0%)

**Table 3:** Agreement analysis by slip grade of SDSG classification

Slip grade	Inter-observer reliability		Intra-observer reproducibility	
	Agreement (%)	K	Agreement (%)	K
All cases	89.2%	0.813	90.4%	0.873
Low-grade slips	85.3%	0.729	89.5%	0.852
High-grade slips	91.2%	0.864	92.7%	0.911

The inter-observer reliability of CARDS classification was substantial at 72.6% (68.4-79.5%) with an associated K value of 0.620 (0.572-0.701) (Table 4), of which 50 (42.7%) cases showed perfect agreement among all 5 observers in the first assessment, while 49 (41.9%) cases in the second assessment. Nevertheless, at least 3 observers showed agreement on 89 (76.1%) cases in the first assessment and 86 (73.5%) cases in the second assessment (Table 5). In addition, the K values of 3 items of CARDS system: disk space height, sagittal vertebral translation, and kyphotic alignment were 0.618, 0.477, 0.725, respectively (Table 6).

**Table 4:** Inter-observer reliability of CARDS classification

Observers^**a**^	Cases in agreement between observers					Agreement (%)	K
	Type A	Type B	Type C	Type D	Total		
1-2	14	38	32	5	89	76.1	0.663
1-3	13	34	30	6	83	70.9	0.597
1-4	14	39	33	7	93	79.5	0.701
1-5	12	33	30	5	80	68.4	0.572
2-3	11	34	31	6	82	70.1	0.589
2-4	12	34	33	5	84	71.8	0.610
2-5	14	32	32	5	83	70.9	0.596
3-4	13	37	33	7	90	76.9	0.673
3-5	12	34	33	6	85	72.6	0.628
4-5	12	33	30	5	80	68.4	0.573
Overall	-	-	-	-	-	72.6	0.620

^**a**^1, 2, 3, 4, 5 represent the 5 observers who participated in the study

**Table 5:** Agreement of CARDS classification among five observers

	The first assessment	The second assessment
**Agreement among all 5 observers**	50 (42.7%)	49 (41.9%)
**Agreement among at least 3 observers**	89 (76.1%)	86 (73.5%)

**Table 6:** Agreement analysis by three items of CARDS classification

Items	Inter-observer reliability		Intra-observer reproducibility	
	Agreement (%)	K	Agreement (%)	K
Disk space height	76.5%	0.618	90.2%	0.762
Vertebral translation	69.2%	0.477	87.6%	0.749
Kyphotic alignment	88.4%	0.725	94.7%	0.855

### Intra-observer reproducibility

Reproducibility analysis of the same observer’s results after 12 weeks showed that the intra-observer agreement of SDSG classification was near perfect at 88.2% (84.6-92.3%) with an average K value of 0.861 (0.823-0.906) (Table 7). The intra-observer agreement of slip grade (low-grade vs. high-grade slips) was near perfect at 90.4% (88.0-93.7%), with a k value of 0.875 (Table 3).

**Table 7:** Intra-observer reproducibility of SDSG classification

Observers^**a**^	Cases in agreement between first and second assessment							Agreement (%)	K
	Type 1	Type 2	Type 3	Type 4	Type 5	Type 6	Total		
1	18	31	22	14	11	7	103	88.0	0.859
2	17	32	22	14	13	7	105	89.7	0.871
3	16	30	23	11	13	6	99	84.6	0.823
4	18	31	20	13	12	7	101	86.3	0.844
5	17	32	23	15	13	8	108	92.3	0.906
Overall	-	-	-	-	-	-	-	88.2	0.861

^a^1, 2, 3, 4, 5 represent the 5 observers who participated in the study

The intra-observer reproducibility of CARDS classification ranged from 87.2 to 94.0% with an average percentage of 90.4%, and the K value was 0.878 (0.835-0.917), which was considered near perfect agreement (Table 8). The K values of 3 items were 0.762, 0.749, 0.855, respectively (Table 6).

**Table 8:** Intra-observer reproducibility of CARDS classification

Observers^a^	Cases in agreement between first and second assessment					Agreement (%)	K
	Type A	Type B	Type C	Type D	Total		
1	18	45	34	7	104	88.9	0.862
2	16	44	35	7	102	87.2	0.835
3	19	46	35	8	108	92.3	0.904
4	20	50	33	7	110	94.0	0.917
5	18	47	32	8	105	89.7	0.874
Overall	-	-	-	-	-	90.4	0.878

^a^1, 2, 3, 4, 5 represent the 5 observers who participated in the study

### Comparison of SDSG and CARDS classifications

SDSG system had better inter-observer reliability in comparison with CARDS system while there was significant difference between the relevant K values of the two classifications (*p* < 0.01). In addition, the two classifications had similar intra-observer reproducibility since there was no significant difference between the K values (*p* > 0.05).

## Discussion

At present, the simplest classification of lumbar DS is Meyerding system [6], which is to grade according to vertebral translation. However, it cannot accurately describe the state and judge the severity of spondylolisthesis to further guide treatment and predict prognosis. Other traditional classification of lumbar DS mainly includes Wiltse and Marchetti classification [13–15]. These classifications have significant defects that they lack quantitative indexes and cannot determine the degree of spondylolisthesis, which makes them difficult to be evaluated and inferior in reproducibility.

The abovementioned classifications all emphasizes on characteristics of slipped vertebrae or bony structures, without considering disk degeneration, spinal-pelvic sagittal balance and clinical symptoms which are regarded as key factors to judge whether lumbar DS will progress [16–18].

SDSG classification gives spine surgeons a clear definition of spinal-pelvic sagittal balance, and helps them to provide targeted treatment for patients [19, 20]. There is always a dispute about whether severe spondylolisthesis needs reduction. According to current study of biomechanics, combined with SDSG classification, specialists have reached a consensus that for patients with imbalanced spine or pelvis, reduction should be emphasized to correct the imbalance as well as the external deformities, and provide a more favorable biomechanical environment for bone graft. For patients with balanced pelvis and spine, either fusion in situ or fusion with reduction and fixation can be used. The results show that the inter- and intra-observer agreement K value of SDSG classification are 0.704 and 0.861, respectively, which are slightly higher than that of the previous agreement studies by Mac-Thiong et al. [21] (0.65, 0.74) and Bao et al. [22] (0.648, 0.830), indicating relatively better consistency strength. In these studies, the case scope of assessment of SDSG classification covered dysplastic, degenerative and isthmic spondylolisthesis. However, to formulate a case inclusion criterion applicable to both classifications, the cases involved in the study were limited as L5/S1 lumbar DS, while narrower and more specific scope often leads to greater reliability, which may be one of the factors that caused the differences in the results. Moreover, it is worth mentioning that the research has even better reliability of intra-observer reproducibility than those previous, for there is an only 1-day or 2-week interval between the 2 acquisition sessions in those studies, while too short interval will make observers in the second assessment tend to evaluate according to their recollections of the first assessment, and thus may reduce the reliability of results. The 12-week interval in our study may be a more appropriate choice. In addition, we analyzed the agreement of slip grade, and the results show that both inter- and intra-observer K values are high (0.813, 0.875), which may be largely attributed to the accurate measurement of computer-assisted technique, and through which can be seen that the slip grade is not the key factor for the deviation of classifying results between observers. Therefore, we believe that low resolution and clarity of radiographs, and serious osteoporosis of elderly patients make it difficult to judge the bone structure and anatomic landmark, which leads to deviations of sagittal parameter measurements.

As a more recent established one, CARDS system can provide a relatively ideal treatment plan for patients in comparison with other classifications. For those without clinical symptoms (type A0), conservative treatment is recommended [18]. On the aspect of surgery, simple decompression can be performed on type A1 and A2 patients [23], while internal fixation and fusion is practical in type B or C patients. For cases of type D, internal fixation is needed to correct kyphosis deformity and interbody fusion cage is needed to reconstruct anterior column support, so that physiological lumbar lordosis and fusion rate can be improved as much as possible [24]. Whether the leg pain exists or not is regarded as the clinical index for subtypes, which is also helpful to guide surgical plan. A study published in recent years has confirmed that patients with leg pain as the main symptom before surgery have better postoperative effect than those with back pain as the main symptom [25]. Compare with the previous study by Kepler et al. [9] and Kong et al. [26], the inter-observer reliability of CARDS system is lower. CARDS system was initially proposed based on L4/5 DS; however, our study applied it to L5/S1 segment. Since the two segments had different structure that L4/5 more tended to the horizontal direction, it would be easier for observers to assess DS [27]. Thus may lead to difference between the results. Another reason may explain this is the relatively larger sample size (117 in ours, 126 in Kepler’s, and 146 in Kong’s), which may reduce the inter-observer deviation and make the result more accurate. In addition, we found that no matter in inter- or intra-observer agreement test, the K values of sagittal vertebral translation were lower than those of disk space and kyphotic alignment, which merely indicates “moderate” agreement. Firstly, the classification requires that any translation longer than 5 mm in neutral, flexion, or extension lateral radiographs should be classified as type C, while in the actual process, observers may have certain marking or measuring deviations. Furthermore, with the multiple measurements, sometimes observers judging by subjective impression is also a factor, which leads to the relatively low agreement.

Both classifications had substantial inter- and intra-observer agreement, while SDSG classification had better inter-observer reliability in comparison with CARDS classification. With regard to sagittal balance parameters, SDSG classification can provide better reference value for surgical strategy. Nevertheless, it does not consider the changes on flexion and extension lateral radiograph, and the evaluation of lumbar instability is insufficient, which reduces the guiding value of surgical treatment, and that is the issue of SDSG classification. Since the clinical symptoms are often the reasons for DS patients to see a doctor, CARDS classification takes leg pain into account, which makes evaluation of scientific and clinical study more convenient, and that is the advantage of CARDS classification. According to the above, CARDS classification is more intuitive and simpler than SDSG classification. However, its morphological types are less and not precise enough, and that will lead to the relatively unclear boundaries between the various types. Therefore, it is more likely to produce deviations when using CARDS classification.

The current study has several limitations. Firstly, is the retrospective design. It is easy to produce selection bias. Secondly, is the relatively small sample size. Expanding the sample population to include non-operative patients of a wider population, allowing for more meaningful statistical testing on the reliability and reproducibility of these parameters. Thirdly, is the relatively low resolution and clarity of radiographs. We believe that it may be more accurate in the practical application to observe high resolution radiograph combining with computed tomography (CT) sagittal reconstruction image. Finally, only L5/S1 single segment DS patients were included in this study, and the agreement of two classifications in other segments and backward slipped DS cases were not discussed. Therefore, in future clinical work, high-quality, multicenter, large sample, and wide case scope studies should be conducted to provide spine surgeons with the best evidence-based information.

## Conclusion

SDSG system had better inter-observer reliability in comparison with CARDS system, and though CARDS system was more intuitive and simpler, it was more likely to produce deviations when using it. Since both SDSG and CARDS systems showed substantial agreement and had great significance in surgical strategy of L5/S1 lumbar DS, they could be widely used in clinical practice. However, we still need more higher-quality, larger samples, and multicenter prospective studies in future work to evaluate whether these classification systems allow better decision-making or prognosis-prediction in individual patients.

## Data Availability

All supporting data can be provided upon request to the authors.

## Abbreviations

DS: Degenerative spondylolisthesis
PI: Pelvic incidence
SS: Sacral slope
PT: Pelvic tilt
SDSG: Spinal Deformity Study Group
CARDS: Clinical and Radiographic Degenerative Spondylolisthesis
K: Kappa
CI: Confidence interval
CT: Computed tomography
